# Fluconazole-induced erythema fixum and edema of the upper lip

**DOI:** 10.1186/2045-7022-4-S3-P87

**Published:** 2014-07-18

**Authors:** Anna Valerieva, Elena Petkova, Maria Staevska, Vasil Dimitrov, Todor Popov

**Affiliations:** 1 Medical University - Sofia, Clinical Centre of Allergology, Clinic of Allergy and Asthma, Bulgaria

## Introduction

Triazole antifungals are commonly used in the treatment of candidiasis. Fluconazole (FCZ) is one of the most frequently prescribed therapy for vaginal fungal infections. Rarely, FCZ has been shown to cause fixed drug eruptions (FDE).

## Case presentation

This is a report of FCZ-induced FDE in a 31-year-old Caucasian woman with recurrent vaginal candidiasis. On 4 consecutive occasions she tried treatment with oral 150mg FCZ and each time skin lesions occurred in the same spots with progressive severity, lasting about 10 days, resulting in a hyperpigmentation, causing suffering and poor self-confidence. Detailed history revealed that the first time she noticed the eruption, there was a single, erythematous plaque on the forehead. On the second and third occasions, this same spot reappeared, but this time accompanied by 2 more similar lesions on both cheeks. The patient was told that this was "mild allergy" and not advised to seek specialized help. She was sent to our university clinic on the 4th occasion when she took a single dose of oral 150mg FCZ (different brand name). The patient had 3 nummular, erythematous plaques: 1 on the forehead (15mm D), 1 in the left and 1 in the right zygomatic areas (15 and 20mm D, respectively). Also, a medium edema in the middle part of her upper lip was present for the first time. The eruptions were characterized by pruritus, stinging and erythema, gradually increasing in intensity from 30min post-ingestion(PI). The edema of the upper lip was slightly painful and occurred 6h PI. We treated the patient with Methylprednisolone 60mg (1 day) and gave her Levocetirizine 5mg for symptomatic relief; as this latter dose did not seem sufficient, we doubled it and this completely suppressed her symptoms. Topical Mometasone was added to the treatment on Day 2. Skin lesions resolved almost completely on Day 7, leaving mild hyperpigmentations. The edema of the lip resolved on Day 3 with no sequel. Histologic evaluation, oral and topical provocation tests with FCZ were not performed because of the delicate facial site of manifestation. Oral provocation test with Itraconazole 100mg was performed and showed to be negative.

## Conclusion

FDE induced by FCZ is not a common occurrence. When it does occur, it can be easily overlooked and misdiagnosed. We rejected cross reactivity with Itraconazole, showing this drug is a viable alternative to FCZ. Oral Levocetirizine at a higher than usual dose helped relieve the subjective symptoms.

